# The Association between Dietary Patterns and Semen Quality in a General Asian Population of 7282 Males

**DOI:** 10.1371/journal.pone.0134224

**Published:** 2015-07-28

**Authors:** Chin-Yu Liu, Yu-Ching Chou, Jane C. -J. Chao, Chien-Yeh Hsu, Tai-Lung Cha, Chih-Wei Tsao

**Affiliations:** 1 Department of Nutritional Science, Fu Jen Catholic University, New Taipei City, Taiwan; 2 School of Public Health, National Defense Medical Center, Taipei, Taiwan; 3 School of Nutrition and Health Sciences, College of Public Health and Nutrition, Taipei Medical University, Taipei, Taiwan; 4 Master Program in Global Health and Development, College of Public Health and Nutrition, Taipei Medical University, Taipei, Taiwan; 5 Nutrition Research Center, Taipei Medical University Hospital, Taipei, Taiwan; 6 Department of Information Management, National Taipei University of Nursing and Health Sciences, Taipei, Taiwan; 7 Division of Urology, Department of Surgery, Tri-Service General Hospital, National Defense Medical Center, Taipei, Taiwan; University Hospital of Münster, GERMANY

## Abstract

**Objective:**

To explore the associations between different dietary patterns and semen quality in a general Asian male population.

**Methods:**

Cross-sectional study. Healthy Taiwanese men aged 18 years or older who participated in a standard medical screening program from 2008-2013 run by a private firm were included in this study. Semen parameters including sperm concentration (SC), total sperm motility (TSM), progressive motility (PRM) and normal sperm morphology (NSM) were recorded. A dietary questionnaire was used to categorize the participants into 5 groups: “Healthy diet”, “Western diet”, “High-carbohydrate diet”, “High sweet snacks & sugar-sweetened drinks” and “High-sodium diet”.

**Results:**

A total of 7282 men completed the questionnaire regarding dietary pattern, and examination of anthropometric indexes was performed and laboratory data were obtained. A high intake of a “Western diet” resulted in statistically linear declines of SC and NSM (P < 0.001 and P < 0.001). Similarly, a greater intake of “High sweet snacks & sugar-sweetened drinks” was associated with a lower SC (P = 0.001). Increased intake of a “High-carbohydrate diet” was related to higher prevalences of abnormal TSM and PRM (P = 0.012 and P = 0.025). Similarly, a greater intake of a “High-sodium diet” was correlated with an elevated prevalence of abnormal NSM (P = 0.035).

**Conclusions:**

This study showed that a greater intake of a “Western diet” is associated with poorer SC and NSM, a “High sweet snacks and sugar-sweetened drinks” intake is correlated with a lower SC, and high-carbohydrate food is related to elevated prevalences of abnormal TSM and PRM.

## Introduction

During the past few decades, there has been much discussion about changes in semen quality, with some studies showing its significant decline [1–3]. Other studies have indicated no significant change in semen quality [4, 5]. Despite the heterogeneity in the study populations, overall, the data indicate a decline in sperm concentration in males of most Western countries according to studies conducted during 1934–1996 [6].

Several studies have indicated not only a decline in sperm concentration in most Western countries, but also poor sperm motility and an abnormal sperm morphology. While genetics, the endocrine system, obesity, age, smoking status and heavy alcohol use are known risk factors for a decreased sperm quality, there is increasing evidence that nutrition could play an important role in influencing semen quality [7–9]. Over the past 50 years, the average Western diet has changed dramatically in the U.S. compared with the 1950s, today’s diet typically consists of higher intakes of total calories, meat, cheese, added fats and sugars, which typically reflect a poorer diet quality.

In addition to some studies that have demonstrated that a “western” diet, which is high in animal foods, processed foods, fats and sugar, might have a negative effect on male fertility, other recent studies have also indicated that a "prudent" diet, in which more fish/seafood, poultry, walnuts, vegetables and whole grain foods are consumed, may benefit sperm quality [9–13]. However, based on the available literature in PubMed, no study has been performed in Asian countries. Therefore, the objective of the present study was to investigate the correlations between different dietary patterns and sperm parameters in a large general Asian population. This was the first study to explore the relationships between dietary patterns and semen quality among a general population of Asians.

## Materials and Methods

### Data source

In this cross-sectional cohort study, the cohort initially consisted of 7941 healthy male individuals aged 18 years or older who participated in a standard medical screening program run by a private firm (MJ Health Management Institution, Taipei, Taiwan) between January 2008 and May 2013. The firm attracted paying participants from all over Taiwan because of its known quality services, operational efficiency, and key facilities that were easily accessible. Membership of the program was required, with discounts in examination fees offered for people with a large-sized family or related individuals and for regular members who came back for repeat examinations in subsequent years, and these incentives succeeded in attracting and sustaining a large number of customers. Each participant signed a consent form authorizing MJ Health Management Institution to process the data generated from medical screening. Ethical reviews (Institutional Review Boards) were processed and approved at MJ Health Management Institution and at Tri-Service General Hospital in Taiwan. Data related to individual identification were removed and remained anonymous during the entire study process.

Semen samples were collected via masturbation following at least 3 days of abstinence using home-collection kits with a sterile plastic container, and the samples were sent to the laboratory for analysis within one hour. Four dependent semen parameters, including sperm concentration (SC), total sperm motility (TSM), progressive motility (PRM) and normal sperm morphology (NSM), were recorded. SC was evaluated using a hemocytometer (Improved Neubauer; Hauser Scientific, Inc., Horsham, PA, USA). Samples were diluted in a solution of 0.6 M NaHCO_3_ and 0.4% (v/v) formaldehyde in distilled water. Sperm motility was classified as PRM (WHO class A+B) and TSM (WHO class A+B+C). Briefly, 10 microliters of well-mixed semen were placed on a clean glass slide that had been kept at 37°C and covered with a 22 × 22 mm coverslip. The slide was placed on the heating stage of a microscope at 37°C and immediately examined at ×400 magnification. Owing to the studied subjects being a general population, the semen parameters of most subjects were within the normal range. In particular, the abnormal NSM rate according to the latest WHO 2010 criteria (< 4%) was too low to enable us to evaluate the trend according to different anthropometric indexes. Therefore, the sperm morphology was assessed using WHO 1999 classification and LIFE data-based study [14], and the abnormal criterion of NSM was defined as < 30% in this study.

In addition, all subjects completed a questionnaire related to dietary pattern and had their biochemical data recorded in order to analyze the relationships between different dietary patterns and semen quality in a large cohort study of the general population. Most food frequency questionnaires have been designed following the principles developed by American researchers, which may not be completely applicable in Taiwan. Therefore, in this study, we used a questionnaire based on the characteristics of Taiwanese dietary patterns according to the results of the Nutrition and Health Survey conducted by the government in Taiwan. This was a computerized questionnaire and was designed to display comprehensive food lists for self-selection by the participants. The questionnaire included the frequency and amount of consumption for each food group. There were 19 items in the questionnaire that covered the major Taiwanese dietary patterns. For analysis, the dietary patterns were then assessed and divided into five groups: “Healthy diet”, “Western diet”, “High-carbohydrate diet”, “High sweet snacks & sugar sweetened drinks” and “High-sodium diet”. The “Healthy diet” contained three different foods in total: light-color vegetables, dark-color vegetables and fruits. The definition of a “Western diet” was seven food groups, including milk, cheese, yogurt, meat, seafood, salad and fried food. The “High-carbohydrate diet” included three foods: refined grains, whole grains and root vegetables. “High sweet snacks & sugar sweetened drinks” included three types of food: cake or cookies, additional sugars and sweetened beverages. The “High-sodium diet” involved three food items: processed canned food, instant noodles and condiments. All items on the questionnaire were scored 1, 2, 3, 4 or 5, representing no intake, less than one exchange/week, one to three exchange(s)/week, four to six exchanges/week, one exchange/day and more than two exchanges/day, respectively. Exchanges are foods that are grouped together into categories or lists according to similarities in nutritional values; for example, meats, starches, dairy, vegetables and fruit. Measured portions of foods within the categories may be exchanged or traded when planning meals. Each serving of a food has about the same amount of carbohydrate, protein, fat, and calories as the other foods on the same list. Mean scores were calculated for all items of each dietary pattern, with higher scores indicating a greater intake of an individual dietary pattern. In this study, Cronbach’s alpha for the overall scale was 0.895 and Cronbach’s alphas for the individual domains ranged from 0.703–0.873.

### Statistical analyses

Owing to the properties of the questionnaire, we applied the quartile method to assess the relationships between intakes of all these food items and semen quality. Differences in the sperm parameters related to various food intake amounts as assessed by the food frequency questionnaire were compared using analysis of variance (ANOVA). To illustrate the real association between semen quality and obesity, all subjects were categorized by quartile according to each anthropometric index, and the severities and prevalences of abnormal sperm parameters were analyzed while increasing the intake of some dietary patterns. Abnormal sperm parameters were defined as SC < 15 M/ml, TSM < 40%, PRM < 32% and NSM < 30%. The frequencies of abnormal sperm parameters in each quartile were compared by the chi-square test. To categorize each dietary pattern, we assessed their relationships with semen outcomes by first conducting a logistic trend analysis and then estimating the odds ratios (ORs). Moreover, the topic of smoking is popular in relation to public health, and therefore we applied a questionnaire to assess the relationship between smoking and male infertility. According to the questionnaire, smoking duration was assessed as “0”: non-smoker, “1”: smoking < 1 year, “2”: 1 year to < 3 years, “3”: 3 years to < 5 years, “4”: 5 years to < 10 years, “5”: 10 years to < 20 years, “6”: 20 years or more. We then explored the association between smoking and semen quality by the quartile method (dividing [“0”], [“1”+”2”], [“3”+”4”], [“5”+”6”]). All analyses were conducted using SPSS statistical software (version 13.0, SPSS Inc, Chicago, IL).

## Results

After excluding those with incomplete records and subjects with a major systemic disease or a history of reproductive organ disorder, a total of 7282 subjects were enrolled in the study. All subjects were aged between eighteen and seventy-five years and the semen quality, including sperm concentration (SC, M/ml), total sperm motility (TSM, %), progressive sperm motility (PRM, %) and normal sperm morphology (NSM, %), was examined.

The mean age of the whole population was 31.75 years, with an average height of 172.21 cm and an average body weight of 70.65 kg. The mean anthropometric indexes, including body mass index (BMI), waist circumference (WC), hip circumference (HC), waist-to-hip ratio (WHR), waist-to-height ratio (WHtR) and body fat percentage, were 23.79 ± 3.30 kg/m^2^, 81.06 ± 8.52 cm, 96.24 ± 6.44 cm, 0.84 ± 0.14, 0.47 ± 0.05 and 23.47 ± 5.49%, respectively (Table 1). We discovered that age, SC and NSM were statistically inversely associated with a “Western diet” intake, even after adjusting for the cofactors. Otherwise, the triglyceride level and all the anthropometric indexes, including BMI, WC, HC, WHR, WHtR and body fat percentage, were significantly positively correlated with a “Western diet” intake. Only SC was statistically inversely correlated with an increasing smoking duration. Although other semen parameters including TSM, PRM and NSM exhibited a reverse trend with each quartile of smoking duration, this was without statistical significance (ANOVA test; P = 0.024; P = 0.245; P = 0.762; P = 0.648; data not shown).

**Table 1:** Characteristics of the general male population with a Western diet (*n* = 7282).

Characteristics	Q1 ≤ 10 (*n* = 2191)	Q2: 11–12 (*n* = 2198)	Q3: 13–14 (*n* = 1695)	Q4 ≥ 15 (*n* = 1198)	Total (n = 7282)	P
**Age (years)**	32.31 (32.10, 32.52)	31.79 (31.60, 31.98)	31.47 (31.27, 31.68)	31.06 (30.82, 31.31)	31.75 (31.64, 31.86)	< 0.001
**Triglyceride**	116.55 (113.36, 119.73)	116.90 (113.40, 120.39)	118.86 (115.30, 122.43)	124.47 (118.96, 129.98)	118.50 (116.61, 120.38)	0.037
**Cholesterol**	189.44 (187.98, 190.90)	190.27 (188.88, 191.65)	189.47 (187.89, 191.04)	191.29 (189.34, 193.25)	190.00 (189.22, 190.78)	0.406
**HDL**	50.48 (49.98, 50.98)	50.37 (49.86, 50.87)	50.00 (49.42, 50.58)	49.93 (49.27, 50.59)	50.24 (49.97, 50.52)	0.459
**LDL**	116.39 (115.07, 117.71)	117.24 (115.97, 118.51)	116.25 (114.79, 117.70)	117.52 (115.75, 119.31)	116.80 (116.09, 117.51)	0.565
**Cholesterol ratio**	3.92 (3.87, 3.97)	3.95 (3.90, 3.99)	3.97 (3.92, 4.02)	4.00 (3.94, 4.06)	3.95 (3.93, 3.98)	0.196
**CRP**	0.21 (0.18, 0.23)	0.21 (0.20, 0.23)	0.22 (0.20, 0.24)	0.23 (0.20, 0.25)	0.21 (0.20, 0.23)	0.715
**Prolactin**	12.74 (12.36, 13.12)	12.88 (12.50, 13.26)	12.41 (12.02, 12.80)	12.46 (12.05, 12.87)	12.66 (12.46, 12.85)	0.284
**BMI (kg/m** ^**2**^ **)**	23.38 (23.24, 23.51)	23.67 (23.54, 23.80)	24.03 (23.86, 24.19)	24.45 (24.26, 24.64)	23.79 (23.72, 23.87)	< 0.001
**Waist circumference**	79.95 (79.60, 80.30)	80.75 (80.40, 81.09)	81.75 (81.33, 82.18)	82.75 (82.26, 83.24)	81.07 (80.87, 81.27)	< 0.001
**Hip circumference**	95.38 (95.12, 95.64)	96.04 (95.79, 96.29)	96.81 (96.49, 97.13)	97.49 (97.08, 97.90)	96.26 (96.11, 96.41)	< 0.001
**WHR**	0.837 (0.835, 0.839)	0.840 (0.838, 0.842)	0.843 (0.841, 0.846)	0.860 (0.840, 0.879)	0.843 (0.840, 0.846)	< 0.001
**WHtR**	0.466 (0.464, 0.468)	0.470 (0.468, 0.472)	0.473 (0.471, 0.476)	0.479 (0.476, 0.481)	0.471 (0.470, 0.472)	< 0.001
**Body fat (%)**	22.80 (22.57, 23.02)	23.35 (23.13, 23.57)	23.83 (23.55, 24.10)	24.38 (24.06, 24.70)	23.46 (23.34, 23.60)	< 0.001
**SC** ^**a**^ **(M/ml)**	53.67 (52.00, 55.33)	53.62 (51.93, 55.30)	52.46 (50.66, 54.26)	51.87 (49.40, 54.35)	53.08 (52.15, 54.00)	< 0.001
**TSM** ^**a**^ **(%)**	65.02 (64.40, 65.64)	65.06 (64.45, 65.68)	64.89 (64.21, 65.58)	65.25 (64.44, 65.06)	65.04 (64.71, 65.38)	0.936
**PRM** ^**a**^ **(%)**	45.67 (45.02, 46.33)	46.19 (45.52, 46.85)	46.30 (45.53, 47.08)	46.92 (46.00, 47.83)	46.18 (45.81, 46.55)	0.185
**NSM** ^**a**^	67.79 (67.16, 68.42)	67.75 (67.13, 68.38)	66.70 (66.01, 67.40)	65.68 (64.83, 66.53)	67.18 (66.84, 67.52)	< 0.001

^a^ After adjustment for age, BMI, waist circumference, hip circumference, body fat and smoking, the P values of SC, TSM, PRM, NSM were <0.001, 0.148, 0.491, <0.001,respectively.

BMI: Body mass Index, HDL: High density lipoprotein, LDL: Low density lipoprotein, Cholesterol Ratio: Cholesterol/HDL, CRP: C-reactive protein, SC: Sperm concentration, TSM: Total sperm motility, PRM: Progressive motility, NSM: Normal sperm morphology; WHR: Waist-hip ratio; WHtR: Waist-to-height ratio.

The results showed that the SC and NSM of each upper quartile for the “Western diet” and “High sweet snacks & sugar sweetened drinks” were lower than those of the lower quartile indexes. The mean SC values of the individuals with a “Western diet” and those with a high intake of “Highly sweet snacks & sugar-sweetened drinks” had significantly negative correlations (ANOVA test, P < 0.001 and P *<* 0.001) with each quartile of the different dietary patterns (Table 2). In the individuals with an intake of a “Western diet” and “Highly sweet snacks & sugar sweetened drinks”, increased quartiles of those showed statistically inverse correlations with NSM (ANOVA test, P *<* 0.001 and P = 0.002).

**Table 2:** Dietary patterns by semen parameters.

Characteristics	Quartile	Concentration (M/ml) median (n = 7282)	% Total motility median (n = 7266)	% Progressive motility median (n = 7254)	% Normal morphology median (n = 7276)
**Healthy diet**	Q1: ≤ 2	52.279 (49.604, 54.955)	65.830 (64.859, 66.801)	46.985 (45.899, 46.071)	67.585 (66.585, 68.585)
	Q2: 3	52.435 (50.805, 54.066)	65.271 (64.665, 65.877)	46.226 (45.561, 46.891)	66.965 (66.353, 67.577)
	Q3: 4	54.336 (52.781, 55.891)	65.127 (64.583, 65.670)	46.476 (45.871, 47.080)	67.360 (66.780, 67.921)
	Q4: ≥ 5	52.640 (50.758, 54.523)	64.450 (63.741, 65.159)	45.344 (46.598, 46.091)	67.139 (66.421, 67.856)
	P, adjusted P	0.288, 0.501	0.124, 0.414	0.054, 0.276	0.692, 0.371
**Western diet**	Q1: ≤ 10	55.669 (52.003, 55.334)	65.022 (64.404, 65.641)	45.674 (45.020, 46.330)	67.791 (67.163, 68.418)
	Q2: 11–12	53.617 (51.932, 55.303)	65.062 (64.445, 65.678)	46.186 (45.518, 46.853)	67.754 (67.131, 68.376)
	Q3: 13–14	52.460 (50.661, 54.258)	64.894 (64.212, 65.577)	46.305 (45.530, 47.079)	66.702 (66.009, 67.395)
	Q4: ≥15	51.875 (49.402, 54.348)	65.248 (64.437, 66.060)	46.915 (46.002, 47.828)	65.677 (64.827, 66.527)
	P, adjusted P	<0.001, <0.001	0.936, 0.148	0.185, 0.491	<0.001, <0.001
**High-carbohydrate diet**	Q1: ≤ 3	52.702 (50.887, 54.516)	65.822 (65.207, 66.436)	45.863 (46.156, 47.571)	66.809 (66.138, 67.480)
	Q2: 4	52.408 (50.888, 53.929)	64.908 (64.345, 65.471)	46.055 (45.448, 46.662)	67.085 (66.528, 67.642)
	Q3: 5	53.898 (51.965, 55.831)	64.797 (64.070, 65.525)	46.142 (45.360, 46.924)	67.651 (66.925, 68.378)
	Q4: ≥ 6	54.387 (52.181, 56.593)	64.814 (64.003, 65.625)	45.586 (44.706, 46.465)	67.507 (66.675, 68.340)
	P, adjusted P	0.404, 0.170	0.101, 0.116	0.149, 0.084	0.325, 0.085
**High sweet snacks & sugar-sweetened drinks**	Q1: ≤ 3	55.741 (53.689, 57.794)	64.864 (64.125, 65.602)	45.933 (45.149, 46.717)	67.509 (66.769, 68.248)
	Q2: 4	53.508 (51.584, 55.433)	64.666 (64.002, 65.330)	45.813 (45.013, 46.612)	67.780 (67.120, 68.478)
	Q3: 5	53.878 (51.832, 55.924)	65.052 (64.330, 65.775)	45.732 (44.997, 46.467)	66.935 (66.186, 67.684)
	Q4: ≥ 6	50.762 (49.290, 52.234)	65.549 (64.978, 65.120)	46.954 (46.325, 47.584)	66.688 (66.106, 67.271)
	P, adjusted P	0.001, <0.001	0.222, 0.522	0.307, 0.081	0.069, 0.002
**High-sodium diet**	Q1: ≤ 4	52.915 (50.520, 55.310)	64.528 (63.660, 65.396)	46.246 (45.322, 47.171)	67.122 (66.276, 67.969)
	Q2: 5	53.593 (49.847, 57.338)	65.267 (64.125, 66.409)	46.478 (45.219, 47.737)	67.036 (65.915, 68.158)
	Q3: 6	51.666 (47.422, 55.910)	64.961 (63.490, 66.433)	46.796 (45.221, 48.371)	66.133 (64.590, 67.676)
	Q4: ≥ 7	49.906 (46.034, 53.779)	66.277 (64.760, 67.794)	48.309 (46.299, 50.119)	66.551 (64.915, 68.187)
	P, adjusted P	0.6, 0.262	0.282, 0.437	0.232, 0.321	0.665, 0.130

Adjusted P: P values obtained after adjustment for age, BMI, waist circumference, hip circumference, body fat and smoking.

To clarify the detailed relationships between different dietary patterns and semen quality, the semen quality as assessed by the prevalence of abnormal sperm parameters was examined by the quartiles of all the above-defined different dietary patterns (Healthy, Western, High-carbohydrate, High sweet snacks & sugar-sweetened drinks, and High-sodium). Our analyses showed that there was a linear association between a greater intake of a “High-carbohydrate diet” and increased prevalences of abnormal TSM (Table 3, < 40%, P = 0.012) and PRM (< 32%, P = 0.025). A “High-sodium diet” intake was associated with a higher prevalence of an abnormal NSM (P = 0.035).

**Table 3:** The associations of abnormal semen quality with dietary patterns.

Characteristics	Quartile	Concentration < 15 M/ml, (%)	Total motility < 40%, (%)	Progressive motility < 32%, (%)	Normal morphology < 30%, (%)
**Healthy diet**	Q1: ≤ 2	9.2	4.4	18.1	0.4
	Q2: 3	10.6	4.7	20.3	0.8
	Q3: 4	9.3	4.7	19.5	1.0
	Q4: ≥ 5	9.4	5.5	20.7	0.7
	P^a^	0.384	0.565	0.409	0.342
**Western diet**	Q1: ≤ 10	9.9	5.0	20.3	0.8
	Q2: 11–12	9.6	5.3	19.7	0.7
	Q3: 13–14	9.4	4.5	20.0	0.5
	Q4: ≥15	10.4	4.7	18.8	1.1
	P^a^	0.846	0.695	0.738	0.293
**High-carbohydrate diet**	Q1: ≤ 3	9.2	3.5	17.5	0.7
	Q2: 4	10.7	4.7	20.5	0.7
	Q3: 5	9.1	5.4	20.2	0.9
	Q4: ≥ 6	9.0	5.5	21.5	0.8
	P^a^	0.171	**0.012**	**0.025**	0.934
**High sweet snacks & sugar sweetened drinks**	Q1: ≤ 3	7.9	5.4	19.4	0.6
	Q2: 4	10.5	5.3	21.7	0.8
	Q3: 5	10.1	4.3	21.6	1.0
	Q4: ≥ 6	10.0	4.5	17.7	0.7
	P^a^	0.063	0.364	0.059	0.636
**High-sodium diet**	Q1: ≤ 4	10.0	5.5	19.6	0.3
	Q2: 5	9.5	3.1	19.8	0.2
	Q3: 6	9.7	5.1	15.5	1.0
	Q4: ≥ 7	9.9	3.2	17.0	1.3
	P^a^	0.992	0.081	0.213	**0.035**

^a^ by the Chi-square test.

In our cohort study, a “High-carbohydrate diet” with the higher quartile of intake had a 1.570~1.604-fold higher odds of a low TSM (Table 4, 95% confidence interval (CI): 1.130–2.183; 95% CI: 1.191–2.159) as compared with the lowest quartile of carbohydrate intake. Similarly, subjects with a “High-carbohydrate diet” had a 1.193~1.288-fold higher odds (95% C.: 1.043–1.411; 95% CI: 1.006–1.415; 95% CI: 1.075–1.543) of an abnormal PSM. Men with a higher quartile of “Highly sweet snacks & sugar-sweetened drinks” intake had a 1.289~1.358-fold higher odds (95% CI: 1.027–1.618; 95% CI: 1.023–1.681; 95% CI: 1.070–1.723) of an abnormal SC as compared with the lowest quartile of the same dietary pattern intake.

**Table 4:** The odds of an abnormal semen quality with dietary patterns.

Characteristics	Quartile	Concentration < 15 M/ml, OR (95% CI)	Total motility < 40%, OR (95% CI)	Progressive motility < 32%, OR (95% CI)	Normal morphology < 30%, OR (95% CI)
**Healthy diet**	Q1: ≤ 2	Reference	Reference	Reference	Reference
	Q2: 3	1.176 (0.897, 1.541)	1.065 (0.726, 1.564)	0.845 (0.683, 1.044)	2.149 (0.628, 7.352)
	Q3: 4	1.015 (0.777, 1.327)	1.070 (0.736, 1.555)	0.972 (0.831, 1.138)	2.681 (0.809, 8.879)
	Q4: ≥ 5	1.028 (0.772, 1.369)	1.256 (1.849, 1.858)	0.928 (0.796, 1.079)	1.854 (0.516, 6.665)
**Western diet**	Q1: ≤ 10	Reference	Reference	Reference	Reference
	Q2: 11–12	0.961 (0.787, 1.173)	1.063 (0.813, 1.391)	0.961 (0.829, 1.115)	0.884 (0.450, 1.739)
	Q3: 13–14	0.943 (0.761, 1.169)	0.896 (0.663, 1.209)	0.982 (0.838, 1.150)	0.572 (0.248, 1.318)
	Q4: ≥15	1.045 (0.828, 1.319)	0.939 (0.675, 1.307)	0.905 (0.757, 1.083)	1.325 (0.647, 2.713)
**High-carbohydrate diet**	Q1: ≤ 3	Reference	Reference	Reference	Reference
	Q2: 4	1.185 (0.972, 1.445)	1.355 (0.944, 1.946)	**1.213 (1.043, 1.411)**	1.046 (0.519, 2.109)
	Q3: 5	0.992 (0.787, 1.251)	**1.570 (1.130, 2.183)**	**1.193 (1.006, 1.415)**	1.254 (0.588, 2.675)
	Q4: ≥ 6	0.979 (0.763, 1.257)	**1.604 (1.191, 2.159)**	**1.288 (1.075, 1.543)**	1.153 (0.504, 2.638)
**High sweet snacks & sugar sweetened drinks**	Q1: ≤ 3	Reference	Reference	Reference	Reference
	Q2: 4	**1.358 (1.070, 1.723)**	0.979 (0.724, 1.325)	1.147 (0.969, 1.357)	1.384 (0.604, 3.171)
	Q3: 5	**1.311 (1.023, 1.681)**	0.793 (0.570, 1.104)	1.140 (0.956, 1.358)	1.641 (0.716, 3.761)
	Q4: ≥ 6	**1.289 (1.027, 1.618)**	0.834 (0.622, 1.116)	0.891 (0.757, 1.049)	1.164 (0.581, 2.619)
**High-sodium diet**	Q1: ≤ 4	Reference	Reference	Reference	Reference
	Q2: 5	1.117 (0.748, 1.668)	1.366 (0.796, 2.345)	1.117 (0.826, 1.511)	1.376 (0.124, 15.215)
	Q3: 6	1.015 (0.667, 1.544)	0.669 (0.352, 1.270)	1.078 (0.789, 1.473)	0.790 (0.049, 12.669)
	Q4: ≥ 7	1.052 (0.705, 1.570)	0.930 (0.525, 1.645)	0.844 (0.619, 1.151)	5.256 (0.655, 42.168)

## Discussion

To our knowledge, the present study was the largest sample size cohort study in a single institute with enrollment of a general population that utilized standardized protocols for a regular medical screening program, including measurement of body size and biochemical studies for the assessment of dietary pattern and semen quality. The study demonstrated that a “Western diet”, “Highly sweet snacks & sugar-sweetened drinks” and “High-carbohydrate diet” were correlated with semen quality.

Previous studies have found that dietary pattern or food groups, different dietary fat food intakes and dairy food are associated with semen quality. Among the studies concerning different general dietary patterns and food groups, Gaskins, Colaci [9] reported that the Prudent pattern diet was positively associated with the percentage of progressively motile sperm, and the Western pattern was not associated with any semen parameter. In addition, studies in Middle-East population by Eslamian et al. [11, 13] stated that a Western diet increased the risk of asthenozoospermia.

Related to recent explorations of the relationship between dairy food and semen quality, Afeiche, Williams [15] clarified that total dairy food intake is inversely related to NSM, and full-fat dairy intake is also associated with a significantly lower PRM among physically active young men, while a low-fat milk intake is particularly related to a higher sperm concentration and progressive motility. In addition, a lower-fat dairy intake is related to a higher SC and PRM in subfertile men, and cheese intake is related to a lower SC among previous or current smokers [16]. The results of these two studies hypothesized that decreased sperm production, manifested as a lower concentration, could be the result of exogenous estrogen from dairy contributing to a negative feedback loop on LH and FSH.

Of the studies discussing different dietary fat food intakes, Attaman, Toth [8] and Jensen, Heitmann [17] found that a higher intake of saturated fat was negatively related to SC and total sperm count; Chavarro, Minguez-Alarcon [18] declared that trans fatty acid intake was inversely related to total sperm count in young healthy men; and Safarinejad and Safarinejad [19] showed that omega-3 supplementation resulted in a higher antioxidant activity in human seminal fluid and enhanced SC, TSM and NSM. Adding walnuts to the Western-style diet has been shown to improve sperm vitality, motility, and morphology, suggesting that omega-6 and omega-3 in walnuts may improve the fatty acid level, which can benefit sperm quality [20].

Our study has demonstrated that fat-rich foods, such as meat and milk, may also decrease the semen quality in humans. Similar study by Mendiola [10] suggesting that a poor semen quality is associated with a higher intake of products incorporating xenobiotics, mainly xenoestrogens or certain anabolic steroids, which are fat-rich substances that can accumulate in fat-rich foods such as meat or milk. Furthermore, the recent results of Attaman’s study [8] were in agreement with the results of a Spanish study showing that intake of processed meat, an important source of saturated fat, is associated with a poorer semen quality [10].

As to “Highly sweet snacks & sugar-sweetened drinks” being associated with a lower SC in our study, several previous studies have also supported this result, noting that a greater sweet intake is correlated with oligoasthenoteratospermia. For example, Eslamian, Amirjannati [11] pointed out that an increased intake of sweet food was significantly associated with a higher risk of asthenozoospermia. Our statistical results concerning the intake of “High-carbohydrate food” being associated with increased prevalences of NSM and PRM are interesting and novel. Carbohydrate-based food is the major food of Asians daily, a dietary pattern quite different from the Western style. Few previous studies have focused on the relationship between carbohydrate intake and semen quality. Only studies by Mendiola et al. and Eslamian et al. have analyzed the association between carbohydrate intake and asthenozoospermia [10, 11]. One argument is that Asian diets may not only be high in carbohydrates, but also in soy bean components, which contain high amounts of phytoestrogens. However, the effect of soy bean components on sperm quality parameters require further study in order to validate their influence. Chavarro et al. [21] studied Caucasian subjects in the main, and found that a higher intake of soy foods is associated with a lower sperm concentration. Whether higher soy bean consumption has deleterious effects on male fertility in Asian men will require further investigation.

No studies have hitherto explored the associations between dietary patterns and semen quality among Asian populations. Regarding disease/cancer incidence and progression, the clinical outcomes are significantly different between Asians and Western populations. Meanwhile, Eastern and Western countries have distinctive lifestyles and dietary patterns. A high-carbohydrate diet is the major food intake in Asian populations. Although it was not found to be associated with any semen parameter in our study, interestingly, a greater intake was correlated with increasing prevalences of abnormal TSM and PRM. Moreover, our findings indicated that a higher intake of a Western dietary pattern was inversely associated with sperm concentration and morphology, a result that is in accordance with neoteric studies [8, 11, 15, 16]. However, the results of our study are in opposition to the conclusion of Gaskins, Colaci [9], which advocated that the Western dietary pattern was not associated with any semen parameter. The probable explanation is that the studies had different definitions and categories of a “Western diet”, resulting in a reverse conclusion.

Furthermore, sweetened beverages and snacks are more popular in the younger generation nowadays, especially teenagers, and this may be a major public health concern. Chiu, Afeiche [22] reported that sugar-sweetened beverage intake was inversely related to sperm motility among young healthy men, an association confined to lean males. Otherwise, the study of Jensen, Swan [23] claimed that a high cola (>14 0.5-L bottles/week) and/or caffeine (>800 mg/day) intake was associated with a reduced sperm concentration and a lower total sperm count, although this was only significant for cola intake. Although consumption of sugar-sweetened beverages has been found to increase insulin resistance in adolescents [24] and adults [25], insulin resistance has been found to increase oxidative stress [26], which in succession would negatively influence sperm motility [27]. However, the dietary pattern of “Highly sweet snacks & sugar-sweetened drinks” in our study was different to that of previous research by Chiu and Jensen.

To our surprise, a healthy diet pattern including vegetables and fruits was not associated with any semen parameter, which is in opposition to the results of many previous studies. Fruits and vegetables are rich in folate, vitamin B6, and antioxidants such as vitamin C, beta carotene and vitamin E, all of which may indirectly improve semen quality, as they have a physiological protection ability against free radicals [28–31]. In addition, a healthy style of diet, such as an intake of more fruits and vegetables, has been suggested to maintain sperm DNA integrity [12]. A plausible alternative explanation could be the presence of environmental contaminants in fruits and vegetables such as pesticides and chlorinated pollutants, which have been found to be associated with a lower semen quality [32, 33]. Therefore, the original anti-oxidative functions of vegetables and fruits could be attenuated by the possible toxic effects of pesticides and pollutants. Furthermore, men exposured to heavy metals in the environment might affect sperm function. For example, men with higher blood mercury levels have been shown to have a high risk of infertility [34], and a high molybdenum level in the blood was found to be associated with a decline in sperm concentration and normal morphology [35].

We recognize that the findings based on the male subjects (mainly Taiwanese males) included in this study may not apply to other populations, particularly if the dietary pattern is dissimilar or foods are produced and processed in different ways. The strengths of this study were its cross-sectional design and the relatively large study sample size of 7282 men. We concluded that a higher “Western diet” intake, including dairy food, meat and saturated trans fatty acid intake, is associated with a poorer SC and NSM. A higher intake of “Highly sweet snacks & sugar-sweetened drinks” is related to a decline in SC. A greater intake of “High-carbohydrate food” is correlated with increased prevalences of abnormal total sperm motility and progressive sperm motility. However, some limitations of our epidemiological study existed, which included the fact that the questionnaire was simply a rough estimation of the connective food groups and was not typical of the food frequency questionnaire (FFQ), and data regarding semen volume and total sperm count were not available due to the protocol of the physical health examination. In addition, no assessments of related endocrine variations were made. Also, the participants had to pay a fee (~US$100) in order to participate in the medical screening program, which, although acceptable for the middle classes of the general population in Taiwan, may exclude poorer populations. In addition, as the participants had only been asked to undertake at least 3 days of abstinence, longer abstinence durations were not recorded in the data obtained from MJ Health Management Institution. Therefore, the effect of abstinence duration could not be examined. Furthermore, as this was a large sample size study, it was difficult to perform on-site collection of semen samples. Therefore, semen samples were collected using home-collection kits. As the semen samples were requested to be sent to the laboratory within one hour, the quality may not be equal to that resulting from on-site collection.

## Conclusions

Intake of a “Western diet” is linked to a poorer SC and NSM, a “Highly sweet snacks and sugar-sweetened drinks” intake is associated with a lower SC, and high-carbohydrate food is related to elevated prevalences of abnormal TSM and PRM. The observed relationships are biologically plausible; nevertheless, data regarding the relationships of diet in general and groups of food in particular with semen quality or male factors of infertility remain limited. Therefore, additional prospective studies, including studies exploring the biological mechanisms, may be needed to explain the associations between dietary patterns and male fertility.

## References

[1] Irvine S, Cawood E, Richardson D, MacDonald E, Aitken J. Evidence of deteriorating semen quality in the United Kingdom: birth cohort study in 577 men in Scotland over 11 years. Bmj. 1996;312(7029):467–71. . 8597676 10.1136/bmj.312.7029.467 PMC2349950

[2] Zou Z, Hu H, Song M, Shen Y, Guo X, McElreavey K, et al Semen quality analysis of military personnel from six geographical areas of the People's Republic of China. Fertility and sterility. 2011;95(6):2018–23, 23 e1–3. Epub 2011/03/30. 10.1016/j.fertnstert.2011.02.052 . 21444069

[3] Rolland M, Le Moal J, Wagner V, Royere D, De Mouzon J. Decline in semen concentration and morphology in a sample of 26,609 men close to general population between 1989 and 2005 in France. Hum Reprod. 2013;28(2):462–70. Epub 2012/12/06. 10.1093/humrep/des415 . 23213178 PMC4042534

[4] Paulsen CA, Berman NG, Wang C. Data from men in greater Seattle area reveals no downward trend in semen quality: further evidence that deterioration of semen quality is not geographically uniform. Fertility and sterility. 1996;65(5):1015–20. . 8612827

[5] Fisch H, Goluboff ET, Olson JH, Feldshuh J, Broder SJ, Barad DH. Semen analyses in 1,283 men from the United States over a 25-year period: no decline in quality. Fertility and sterility. 1996;65(5):1009–14. Epub 1996/05/01. . 8612826 10.1016/s0015-0282(16)58278-8

[6] Swan SH, Elkin EP, Fenster L. The question of declining sperm density revisited: an analysis of 101 studies published 1934–1996. Environmental health perspectives. 2000;108(10):961–6. . 11049816 10.1289/ehp.00108961 PMC1240129

[7] Wong WY, Thomas CM, Merkus JM, Zielhuis GA, Steegers-Theunissen RP. Male factor subfertility: possible causes and the impact of nutritional factors. Fertility and sterility. 2000;73(3):435–42. Epub 2000/02/26. . 10688992 10.1016/s0015-0282(99)00551-8

[8] Attaman JA, Toth TL, Furtado J, Campos H, Hauser R, Chavarro JE. Dietary fat and semen quality among men attending a fertility clinic. Hum Reprod. 2012;27(5):1466–74. Epub 2012/03/15. 10.1093/humrep/des065 22416013 PMC3329193

[9] Gaskins AJ, Colaci DS, Mendiola J, Swan SH, Chavarro JE. Dietary patterns and semen quality in young men. Hum Reprod. 2012;27(10):2899–907. Epub 2012/08/14. 10.1093/humrep/des298 22888168 PMC3442634

[10] Mendiola J, Torres-Cantero AM, Moreno-Grau JM, Ten J, Roca M, Moreno-Grau S, et al Food intake and its relationship with semen quality: a case-control study. Fertility and sterility. 2009;91(3):812–8. Epub 2008/03/04. 10.1016/j.fertnstert.2008.01.020 . 18314116

[11] Eslamian G, Amirjannati N, Rashidkhani B, Sadeghi M-R, Hekmatdoost A. Intake of food groups and idiopathic asthenozoospermia: a case-control study. Hum Reprod. 2012;27(11):3328–36. 10.1093/humrep/des311 22940769

[12] Vujkovic M, de Vries JH, Dohle GR, Bonsel GJ, Lindemans J, Macklon NS, et al Associations between dietary patterns and semen quality in men undergoing IVF/ICSI treatment. Hum Reprod. 2009;24(6):1304–12. 10.1093/humrep/dep024 . 19228759

[13] Eslamian G, Amirjannati N, Rashidkhani B, Sadeghi MR, Baghestani AR, Hekmatdoost A. Adherence to the Western Pattern Is Potentially an Unfavorable Indicator of Asthenozoospermia Risk: A Case-Control Study. Journal of the American College of Nutrition. 2015:1–9. 10.1080/07315724.2014.936983 . 25764357

[14] Eisenberg ML, Kim S, Chen Z, Sundaram R, Schisterman EF, Buck Louis GM. The relationship between male BMI and waist circumference on semen quality: data from the LIFE study. Hum Reprod. 2014;29(2):193–200. 10.1093/humrep/det428 24306102 PMC3896223

[15] Afeiche M, Williams PL, Mendiola J, Gaskins AJ, Jorgensen N, Swan SH, et al Dairy food intake in relation to semen quality and reproductive hormone levels among physically active young men. Hum Reprod. 2013;28(8):2265–75. Epub 2013/05/15. 10.1093/humrep/det133 23670169 PMC3712661

[16] Afeiche M, Bridges ND, Williams PL, Gaskins AJ, Tanrikut C, Petrozza JC, et al Dairy intake and semen quality among men attending a fertility clinic. Fertility and sterility. 2014;101:1280–7. 10.1016/j.fertnstert.2014.02.003 24636397 PMC4008690

[17] Jensen TK, Heitmann BL, Jensen MB, Halldorsson TI, Andersson AM, Skakkebaek NE, et al High dietary intake of saturated fat is associated with reduced semen quality among 701 young Danish men from the general population. The American journal of clinical nutrition. 2013;97(2):411–8. Epub 2012/12/28. 10.3945/ajcn.112.042432 . 23269819

[18] Chavarro JE, Minguez-Alarcon L, Mendiola J, Cutillas-Tolin A, Lopez-Espin JJ, Torres-Cantero AM. Trans fatty acid intake is inversely related to total sperm count in young healthy men. Hum Reprod. 2014;29(3):429–40. Epub 2014/01/15. 10.1093/humrep/det464 24419496 PMC3923511

[19] Safarinejad MR, Safarinejad S. The roles of omega-3 and omega-6 fatty acids in idiopathic male infertility. Asian journal of andrology. 2012;14(4):514–5. Epub 2012/06/05. 10.1038/aja.2012.46 22659579 PMC3720081

[20] Robbins WA, Xun L, FitzGerald LZ, Esguerra S, Henning SM, Carpenter CL. Walnuts improve semen quality in men consuming a Western-style diet: randomized control dietary intervention trial. Biology of reproduction. 2012;87(4):101 10.1095/biolreprod.112.101634 . 22895856

[21] Chavarro JE, Toth TL, Sadio SM, Hauser R. Soy food and isoflavone intake in relation to semen quality parameters among men from an infertility clinic. Hum Reprod. 2008;23(11):2584–90. 10.1093/humrep/den243 18650557 PMC2721724

[22] Chiu YH, Afeiche MC, Gaskins AJ, Williams PL, Mendiola J, Jorgensen N, et al Sugar-sweetened beverage intake in relation to semen quality and reproductive hormone levels in young men. Hum Reprod. 2014;29:1575–84. Epub 2014/05/09. 10.1093/humrep/deu102 . 24812311 PMC4168308

[23] Jensen TK, Swan SH, Skakkebaek NE, Rasmussen S, Jorgensen N. Caffeine intake and semen quality in a population of 2,554 young Danish men. American journal of epidemiology. 2010;171(8):883–91. Epub 2010/03/27. 10.1093/aje/kwq007 . 20338976

[24] Kondaki K, Grammatikaki E, Jimenez-Pavon D, De Henauw S, Gonzalez-Gross M, Sjostrom M, et al Daily sugar-sweetened beverage consumption and insulin resistance in European adolescents: the HELENA (Healthy Lifestyle in Europe by Nutrition in Adolescence) Study. Public health nutrition. 2013;16(3):479–86. Epub 2012/09/27. 10.1017/S1368980012002613 . 23009737 PMC10271790

[25] Stanhope KL, Schwarz JM, Keim NL, Griffen SC, Bremer AA, Graham JL, et al Consuming fructose-sweetened, not glucose-sweetened, beverages increases visceral adiposity and lipids and decreases insulin sensitivity in overweight/obese humans. The Journal of clinical investigation. 2009;119(5):1322–34. Epub 2009/04/22. 10.1172/JCI37385 19381015 PMC2673878

[26] Park K, Gross M, Lee DH, Holvoet P, Himes JH, Shikany JM, et al Oxidative stress and insulin resistance: the coronary artery risk development in young adults study. Diabetes care. 2009;32(7):1302–7. Epub 2009/04/25. 10.2337/dc09-0259 19389821 PMC2699736

[27] Chen SJ, Allam JP, Duan YG, Haidl G. Influence of reactive oxygen species on human sperm functions and fertilizing capacity including therapeutical approaches. Archives of gynecology and obstetrics. 2013;288(1):191–9. Epub 2013/04/02. 10.1007/s00404-013-2801-4 . 23543240

[28] Akmal M, Qadri JQ, Al-Waili NS, Thangal S, Haq A, Saloom KY. Improvement in human semen quality after oral supplementation of vitamin C. Journal of medicinal food. 2006;9(3):440–2. . 17004914 10.1089/jmf.2006.9.440

[29] Eskenazi B, Kidd SA, Marks AR, Sloter E, Block G, Wyrobek AJ. Antioxidant intake is associated with semen quality in healthy men. Hum Reprod. 2005;20(4):1006–12. . 15665024 10.1093/humrep/deh725

[30] Keskes-Ammar L, Feki-Chakroun N, Rebai T, Sahnoun Z, Ghozzi H, Hammami S, et al Sperm oxidative stress and the effect of an oral vitamin E and selenium supplement on semen quality in infertile men. Archives of andrology. 2003;49(2):83–94. Epub 2003/03/08. . 12623744 10.1080/01485010390129269

[31] Mendiola J, Torres-Cantero AM, Vioque J, Moreno-Grau JM, Ten J, Roca M, et al A low intake of antioxidant nutrients is associated with poor semen quality in patients attending fertility clinics. Fertility and sterility. 2010;93(4):1128–33. Epub 2009/01/17. 10.1016/j.fertnstert.2008.10.075 . 19147135

[32] Meeker JD, Hauser R. Exposure to polychlorinated biphenyls (PCBs) and male reproduction. Systems biology in reproductive medicine. 2010;56(2):122–31. Epub 2010/04/10. 10.3109/19396360903443658 . 20377311

[33] Rozati R, Reddy PP, Reddanna P, Mujtaba R. Role of environmental estrogens in the deterioration of male factor fertility. Fertility and sterility. 2002;78(6):1187–94. . 12477510 10.1016/s0015-0282(02)04389-3

[34] Choy CM, Lam CW, Cheung LT, Briton-Jones CM, Cheung LP, Haines CJ. Infertility, blood mercury concentrations and dietary seafood consumption: a case-control study. BJOG: an international journal of obstetrics and gynaecology. 2002;109(10):1121–5. . 12387464 10.1111/j.1471-0528.2002.02084.x

[35] Meeker JD, Rossano MG, Protas B, Diamond MP, Puscheck E, Daly D, et al Cadmium, lead, and other metals in relation to semen quality: human evidence for molybdenum as a male reproductive toxicant. Environmental health perspectives. 2008;116(11):1473–9. 10.1289/ehp.11490 19057699 PMC2592266

